# PTEN Plays an Important Role in Thrombin-Mediated Lung Cancer Cell Functions

**DOI:** 10.1155/2015/459170

**Published:** 2015-03-11

**Authors:** Zhishan Xu, Lingyun Zhu, Min Yao, Genshen Zhong, Qiaoyan Dong, Aiping Yu

**Affiliations:** ^1^Department of Experimental Hematology, Beijing Institute of Radiation Medicine, 27 Taiping Road, Beijing 100850, China; ^2^The First Affiliated Hospital, Xinxiang Medical University, Xinxiang, Henan 453100, China; ^3^The Medical Library of the Chinese PLA, Beijing 100039, China

## Abstract

Thrombin and its membrane receptor, protease-activated receptor 1 (PAR1), have been reported to promote the development of lung cancer *in vitro* and *in vivo*. However, the intracellular molecular mechanism or signaling pathway that mediates the cytological effects after the thrombin-receptor interaction is poorly understood. Our previous study observed that the expression of phosphatase and tensin homolog deleted on chromosome 10 (PTEN) was downregulated in thrombin-stimulated lung cancer. In this study, the role of PTEN in thrombin-mediated cell function and the corresponding cell signaling pathway were studied in lung cancer cell Glc-82. The results indicated that thrombin downregulates the PTEN expression level and that PTEN plays an important role in thrombin-mediated Glc-82 functions, including cell cycle progression, cell apoptosis, and cell migration. The PI3K/AKT signaling pathway and its related proteins, including p27 and S phase kinase associated protein 2 (Skp2), are involved in the effects induced by PTEN downregulation. PAR1 plays a role in thrombin-mediated reduction of PTEN expression. This study suggested that the PTEN/PI3K/AKT signaling pathway plays an important role in thrombin/PAR1-mediated lung cancer cell growth and migration.

## 1. Introduction

Thrombin, beside its pivotal role in hemostasis, plays an important role in tumor growth and metastasis [1]. Thrombin exerts a variety of cellular effects, such as activating platelet-tumor adhesion, tumor growth, metastasis, apoptosis, and tumor-associated angiogenesis [2–4]. Previous studies indicated that thrombin promotes cancer growth and metastasis mainly through the induction of a variety of proangiogenic and prometastatic growth factors [5]. Thrombin promotes the proliferation, adhesion, and migration of endothelial cells, resulting in the angiogenesis in tumor and then the tumor growth and metastasis [6–8]. Recent studies have shown that thrombin can directly stimulate tumor cells. Thrombin promotes cancer cell adhesion, migration, and cell cycle progression [9, 10]. Protease-activated receptor 1 (PAR1) is the major membrane receptor of thrombin [11]. PAR1 is a G-protein-coupled receptor, which is activated by thrombin through the cleavage of the extracellular NH2-terminal domain between the arginine-41 and serine-42 peptide bonds [12]. PAR1 is expressed on endothelial cells and cancer cells, and its expression level is associated with the metastatic ability of a tumor. However, the precise mechanism has not yet been determined; for example, the intracellular molecular mechanism or signaling pathway that mediates the cytological effects after the thrombin-PAR1 interaction is poorly understood.

Our previous study showed that thrombin plays an important role in the growth and metastasis of transplanted lung cancer cell tumors in nude mice. Intriguingly, the PTEN standing for tumor suppressor, phosphatase, and tensin homolog deleted on chromosome 10 (PTEN) was downregulated in the tumor [13]. Mutation or deletion of PTEN frequently results in tumorigenesis, including primary glioblastomas, breast cancer, and lung cancer [14–16]. PTEN is a major negative regulator of the PI-3 kinase (PI3K) by virtue of its PIP3 phosphatase activity, resulting in the attenuation of the downstream signaling of PI3K [17]. AKT is a major downstream target of PI3K for regulating cell survival, cell cycle progression, migration, proliferation, metabolism, tumor growth, and angiogenesis [18, 19]. Downstream substrates of AKT include the FoxO transcription factors, GSK3, BAD, p27, Skp2, and p21, which have important roles in the regulation of the cell cycle, cell growth, migration, and apoptosis [20–22]. Thrombin induces tumor cell cycle activation and spontaneous growth by downregulating p27 and upregulating Skp2 [9]. In addition, PTEN is involved in the regulation of AKT phosphorylation and the expressions of p27 and Skp2 [23, 24]. Therefore, we hypothesized that PTEN, PI3K/AKT, and their corresponding intracellular interactors, p27 and Skp2, participate in thrombin-mediated lung cancer growth or metastasis.

In this study, we initially analyzed the effect of thrombin on the expression of PTEN in different cancer cell lines and found that PTEN was significantly downregulated. We then evaluated the effect of thrombin-induced PTEN downregulation on the regulation of PI3K/AKT signaling pathway and the corresponding cell function in lung cancer Glc-82 cells. The role of PAR1 in thrombin-mediated PTEN downregulation was also evaluated.

## 2. Materials and Methods

### 2.1. Cell Culture

Cell lines Glc-82, PLA-801D, A549 and MCF-7 were cultured in RPMI 1640 medium containing 10% fetal calf serum. Cells lines PLA-801C, MDA231, PC3, PC3M, SMMC-7721, HepG2, and L02 were cultured in DMEM medium containing 10% fetal calf serum. All the above cells were cultured in an incubator at saturated humidity, 37°C, and 5% CO_2_.

### 2.2. Cell Transfection and Cell Sorting

Glc-82 cells were grown to 80% confluence and then transfected with PTEN-shRNA or nontargeting shRNA as a control (Mock) (OriGene Technologies, USA). Transfection was performed using 10 *μ*L of Lipofectamine 2000 (Invitrogen, USA) conjugated with 4 *μ*g of the above plasmid DNA. At 4 h after transfection, the medium was replaced with fresh RPMI 1640 medium containing 10% fetal calf serum and incubation was continued for 48 h. Thereafter, the selective RPMI 1640 medium (containing 10% fetal calf serum and 1.0 *μ*g/mL puromycin) was added. Glc-82 cells were also transfected with PIRES2-EGFP or PIRES2-EGFP-PAR1 overexpression vectors using Lipofectamine 2000 and selected using 500 *μ*g/mL G418 (Invitrogen, USA). To obtain positive transfected cells, 1 × 10^6^ cells were subjected to fluorescence activated cell sorting (FACS) on a flow cytometer (BD, Bioscience).

### 2.3. Reverse Transcription Polymerase Chain Reaction (RT-PCR)

Total RNA was extracted from Glc-82 cells using the Trizol reagent (Tiangen Biotech, China). First-strand cDNA was synthesized using a Trans Script First-Strand cDNA Synthesis Super Mix (TransGen Biotech, China), according to the manufacturer's protocol. cDNA samples were then subjected to PCR amplification. The primers for human PTEN gene were F: 5′-CAA TGA CAG CCA TCA TCA AAG AG-3′ (sense) and R: 5′-GCT CAG ACT TTT GTA ATT TGT G-3′ (antisense). The PCR program was as follows: 95°C for 5 min; 30 cycles of 45 s denaturation at 95°C, 45 s reannealing at 55°C, and 75 s extension at 72°C; and a final extension at 72°C for 10 min. The amplification product was tested by electrophoresis through a 1.0% agarose gel and by nucleotide sequence analysis.

### 2.4. Cell Cycle Phase Assay

Cell cycle phase analysis was determined by flow cytometry (BD, Bioscience). Glc-82 cells in the logarithmic growth phase were starved for 24 h in serum-free RPMI 1640 medium. Thrombin (0.5 U/mL) (Sigma-Aldrich, USA) was added to the medium and incubation continued for 12 h. Thereafter, 1 × 10^6^ cells were centrifuged, immobilized with ice-cold 70% ethanol at 4°C, washed twice with PBS, perforated with 0.1% Triton x-100 for 10 min at room temperature, and then stained with PI (5 *μ*g/mL) containing 100 *μ*g/mL RNase A for 20 min in the dark. Finally, the samples were evaluated by flow cytometry.

### 2.5. Cell Apoptosis Assay

Glc-82 cells in the logarithmic growth phase were starved for 48 h in serum-free RPMI 1640 medium. Thrombin was added and incubated for further 96 h. The cells were then collected by trypsinization and rinsed with PBS. The cells were resuspended in 500 *μ*L of reaction binding buffer, and then 5 *μ*L AnnexinV-APC and 5 *μ*L 7-AAD (KeyGEN Biotech, China) were added, according to the manufacturer's instructions. After complete mixing, the cells were incubated for 15 min in the dark at room temperature and analyzed on the flow cytometer within 1 h after staining. The data obtained was analyzed using CellQuest software.

### 2.6. Cell Migration

Glc-82 cells were grown to 80% confluence and starved for 12 h in serum-free medium before being plated into 24 well transwell plates (8.0 *μ*m pore filters) (Corning, NY). In brief, cells were digested and seeded into the upper chamber at a density of 1 × 10^5^ cells/well in serum-free RPMI 1640 medium with or without thrombin (0.5 U/mL). Cells were allowed to migrate for 20 h at 37°C in a saturated humidity, 37°C, and 5% CO_2_ incubator. The cells were fixed in 600 *μ*L 4% paraformaldehyde for 40 minutes and stained with 2% crystal violet (dilution 1 : 400) for 10 minutes. After washing with PBS, the surface of the upper side was swabbed with a cotton-tipped applicator to remove mechanically nonmigrating cells, and the adherent cells on the lower side of the filter were viewed under a light microscope. Three different visual fields were randomly selected to count the cells and calculate the average migration rate.

### 2.7. Western Blotting

Cells were washed twice with ice-cold PBS and then harvested by scraping the dish in 100 *μ*L RIPA buffer on ice. To evaluate the effects of thrombin on the cells, cells in the logarithmic growth phase were starved for 24 h in serum-free medium before being treated with thrombin (0.5 U/mL, 8 h). The protein concentration was determined using the BCA Protein Assay Reagent (Pierce, Rockford, IL). 40 *μ*g of total protein extract in 25 *μ*L was subjected to sodium dodecyl sulfate–polyacrylamide gel electrophoresis (SDS–PAGE). After SDS-PAGE, the proteins were transferred to a PVDF membrane (Millipore, Billerica, MA). The membrane was blocked in 10% skim milk in TBS with 0.1% Tween (TBS-T) for 2 h at room temperature with gentle rocking. The membrane was then incubated with the appropriate primary antibody in 10% skim milk-containing TBS-T, overnight at 4°C. After washing with TBS-T, the membrane was incubated with the corresponding horseradish peroxidase- (HRP-) conjugated secondary antibody for 45 min at room temperature, with gentle rocking. After extensive washing in TBS-T, the immunoreactive proteins on the blots were visualized using the Pro-light HRP kit (TIANGEN, China). The primary antibodies, including rabbit anti-human PTEN, rabbit anti-phospho-AKT-Ser-473, rabbit anti-Skp2, rabbit anti-p27, and monoclonal primary mouse anti-human GAPDH, were purchased from Cell Signaling Technology (CST, USA). Goat anti-AKT and rabbit anti-PAR1 were purchased from Santa Cruz (USA). Anti-horseradish peroxidase- (HRP-) coupled secondary goat anti-mouse/rabbit and rabbit anti-goat antibodies were purchased from Jackson (USA).

### 2.8. Statistical Analysis

All experiments were repeated at least three times. Data are shown as mean ± standard deviation (SD). Statistical significance was assessed by a two-tailed unpaired Student's* t*-test. A *P* value < 0.05 was considered to be statistically significant.

## 3. Results and Discussion

Thrombin and PAR1 have important roles in cancer growth and metastasis. However, the downstream intracellular mechanism remains unknown. PTEN, a known tumor suppressor, plays a pivotal role in many tumorigeneses. However, whether PTEN is involved in thrombin/PAR1 mediated tumor progression is not fully determined. In our previous study, lung cancer cells Glc-82 were treated with thrombin and transplanted into nude mice. The results revealed that thrombin promoted the growth of the transplanted tumor and metastasis in lung. In addition, the expression of PTEN in the tumor tissue was markedly decreased in the thrombin-treated mice group [9]. In this study, the role of PTEN and its corresponding signaling pathway in thrombin-mediated Glc-82 cells function were further studied.

### 3.1. Thrombin Downregulates PTEN Expression

**Figure 1:**
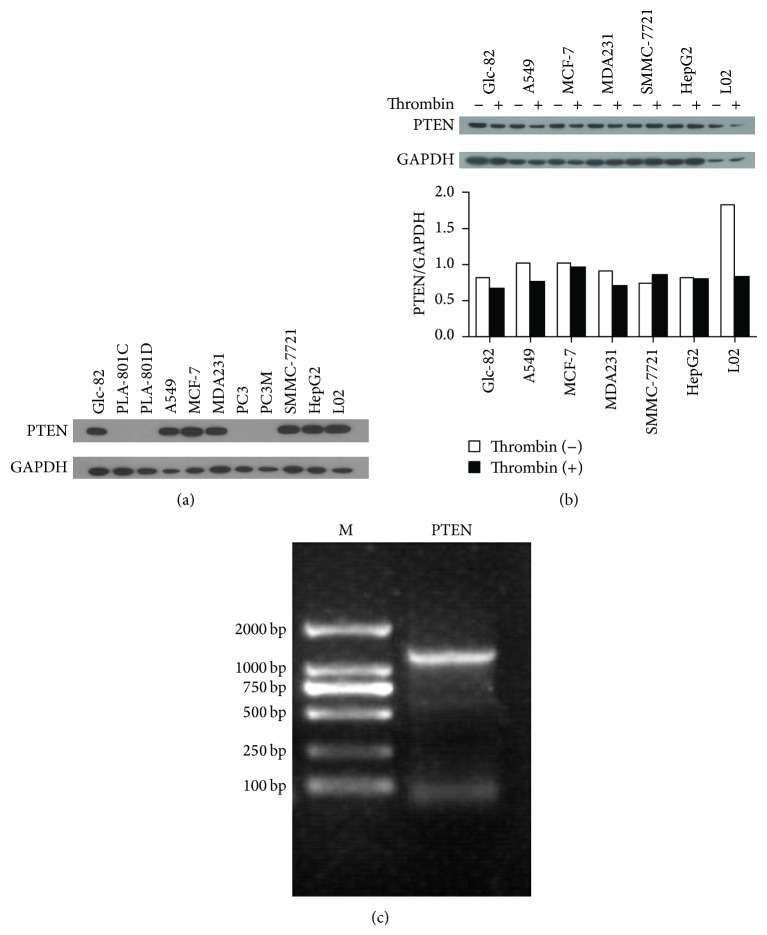
(a) PTEN expression in different cell lines (including 10 cancer cell lines and one normal cell line). (b) The effect of thrombin (0.5 U/mL) on PTEN expression in different cell lines treated for 8 h (including six cancer cell lines and one normal cell line). (c) Agarose gel electrophoresis of the RT-PCR product of PTEN in Glc-82 cells. M: DNA DL2000 marker.

To illustrate the effect of thrombin on PTEN expression, the background protein level of PTEN in 11 cell lines was first detected. As shown in Figure 1(a), in the 11 cell lines, PTEN was expressed in six cancer cell lines and one normal cell line. Treatment with thrombin decreased the PTEN expression level in six out of the seven cells lines (Figure 1(b)). These results indicated that the downregulation of PTEN expression induced by thrombin might be a general biological response.

Our previous study revealed that thrombin promoted lung cancer development* in vivo* and decreased PTEN protein level in tumor tissue; therefore, we were interested in the precise role of PTEN in thrombin-mediated lung cancer cell function. The present study showed that PTEN was expressed in two lung cancer cell lines, A549 and Glc-82 cells, and could be downregulated by thrombin in the both cell lines (Figures 1(a) and 1(b)). Another tumor suppressor, p53, was expressed in A549 in wild-type and can be also downregulated by thrombin. However, p53 gene expression was not detected in Glc-82 cells (data not shown). To avoid the interference of p53 on the evaluation of PTEN effect in thrombin-mediated lung cancer function, Glc-82 cells were chosen as the lung cancer cell model in this study. To analyze whether the PTEN gene is mutated in Glc-82 cells, RT-PCR and nucleotide sequence analysis were performed. The results showed that the PTEN in Glc-82 cells was the wild-type* Homo sapiens* PTEN (as deduced from a search of GenBank) (Figure 1(c)).

### 3.2. Thrombin Decreases PTEN Expression and Activates the PI3K/AKT Signaling Pathway in Glc-82 Cells

**Figure 2:**
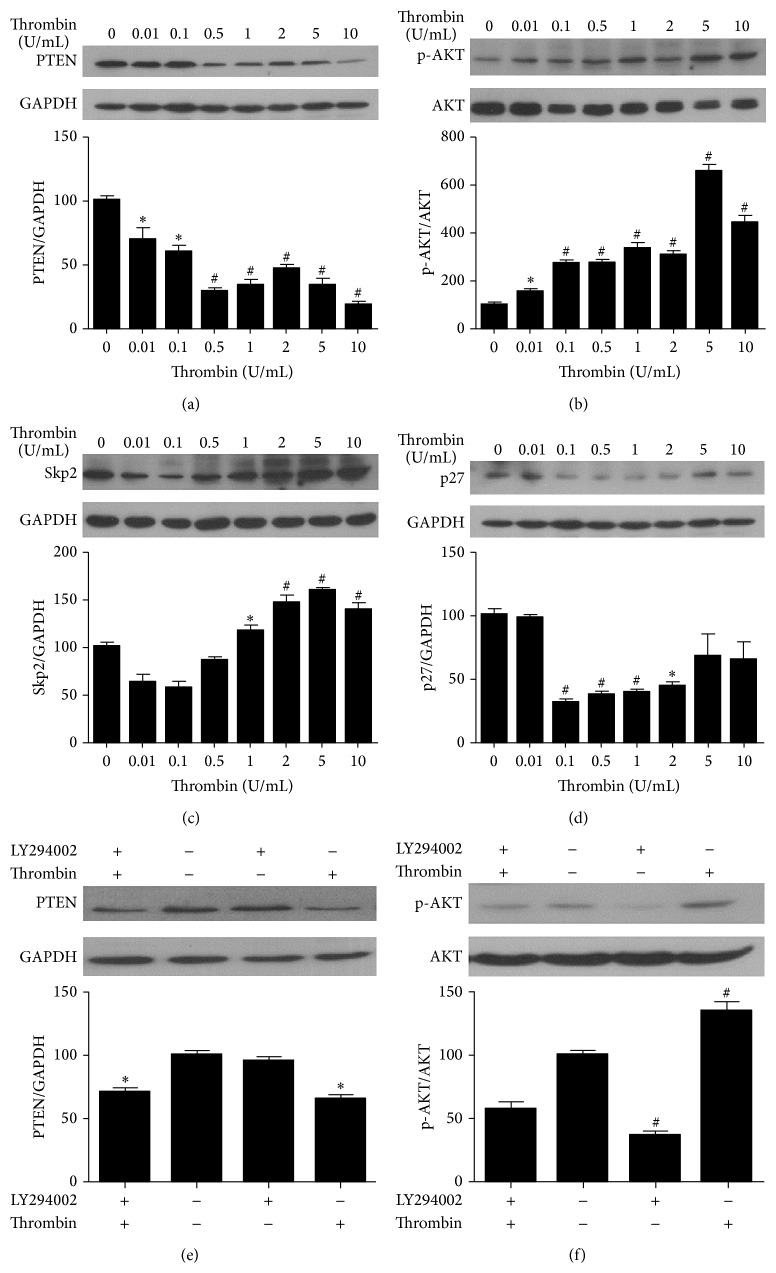
The effects of thrombin on PTEN expression and the PI3K/AKT signaling pathway. Glc-82 cells were starved for 24 h in serum-free medium before treatment with thrombin (0.5 U/mL) for 8 h, with or without LY-294002 (50 *μ*M). Total protein extracts were analyzed for the protein levels of PTEN (a), p-AKT (b), Skp2 (c), and p27 (d) by western blotting. (e) Effect of the PI3K inhibitor LY-294002 and thrombin on PTEN protein expression. (f) Effect of LY-294002 and thrombin on p-AKT. GAPDH and AKT were analyzed as internal loading controls. ^*^ *P* < 0.05 and ^#^ *P* < 0.01, compared with thrombin (0 U/mL).

To identify the effect of thrombin on PTEN and the corresponding signaling pathway, we detected the expression of PTEN and the levels of proteins involved in the PI3K/AKT signaling pathway in Glc-82 cells. The results showed that thrombin markedly decreased the PTEN protein level in a dose-dependent manner (Figure 2(a)). AKT phosphorylation and Skp2 expression increased (Figures 2(b) and 2(c)), while the protein levels of p27 decreased (Figure 2(d)). This is in keeping with a previous report that PTEN acts as a negative regulator of the Skp2 pathway, which is normally used to control S phase entry through the regulation of p27 [25]. Our results further indicated that Skp2, p27, and PTEN together play an important role in the progression of lung cancer. To further determine if the phosphorylation of AKT induced by thrombin was dependent on the activation of the PI3K/AKT pathway, the PI3K inhibitor LY-294002 was used. The results showed that thrombin decreased PTEN expression but that the PI3K inhibitor LY-294002 did not affect the thrombin-induced decrease in PTEN expression (Figure 2(e)). Thrombin (0.5 U/mL) eliminated the inhibitory effect of LY294002 on the PI3K/AKT signaling pathway partially (Figure 2(f)). The above results indicated that thrombin could downregulate PTEN expression and activate the PI3K/AKT signaling pathway in Glc-82 cells.

### 3.3. PTEN Plays a Role in Thrombin-Mediated Activation of the PI3K/AKT Signaling Pathway

To further investigate whether the activation of PI3K/AKT signaling pathway by thrombin is dependent on PTEN expression, a recombinant plasmid expressing a human PTEN specific shRNA (PTEN–shRNA) was transfected into Glc-82 cells. The positive transfected cells were sorted by FACS, and the stable cell lines were identified by puromycin screening.

**Figure 3:**
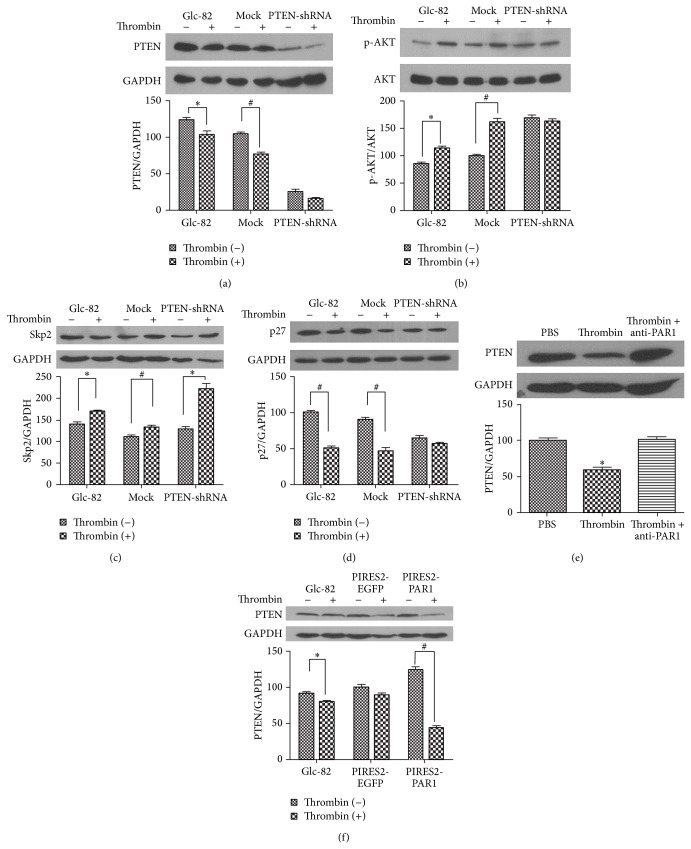
PTEN plays a role in thrombin-mediated activation of the PI3K/AKT signaling pathway. Glc-82, Mock, and Glc-PTEN-shRNA cells were starved for 24 h in serum-free medium before treatment without or with thrombin (0.5 U/mL) for 8 h. The protein levels of PTEN (a), p-AKT (b), Skp2 (c), and p27 (d) were analyzed by western blotting. (e) An anti-PAR1 antibody (0.5 ng/mL) blocked thrombin-mediated PTEN downregulation. (f) The overexpression of PAR1 enhanced the decrease of PTEN expression mediated by thrombin. ^*^ *P* < 0.05 and ^#^ *P* < 0.01, compared with thrombin (0 U/mL).

Figure 3(a) shows that when PTEN was effectively interfered within Glc-PTEN-shRNA cells, thrombin was still able to downregulate PTEN expression, accompanied with an increase of Skp2 expression (Figure 3(b)). Furthermore, the suppression of PTEN expression resulted in increased p-AKT and thrombin did not change the increase in p-AKT (Figure 3(c)). PTEN shRNA interference maintained the decreased level of p27 after thrombin treatment (Figure 3(d)). These results are consistent with reports that thrombin induces the activation of the cancer cell cycle by downregulating p27 expression and inducing Skp2 expression [26]. The shRNA experiments against PTEN indicated that significant increases p-AKT in thrombin-treated Glc-82 cells, further supporting the idea that PTEN is involved in the PI3K/AKT signaling pathway regulated by thrombin in Glc-82 cells.

Thrombin-induced tumor processes are mainly mediated by PAR1, which promotes phosphorylation of the cytoplasmic domain of tissue factor (TF) in enterocytes [27]. In addition, a recent study reported that high TF expression was negatively correlated with the expression of the tumor-suppressor PTEN [28]. We studied the effect of PAR1 on thrombin-mediated suppression of PTEN. As shown in Figure 3(e), thrombin inhibited the expression of PTEN, and an antibody recognizing PAR1 (anti-PAR1) reversed the role of thrombin. To further illustrate the role of PAR1 in thrombin-mediated PTEN downregulation, we constructed an overexpression recombinant plasmid PIRES-PAR1 and transfected the plasmid into Glc-82 cells. The results showed that thrombin (0.5 U/mL) inhibited PTEN expression more effectively when PAR1 was overexpressed (Figure 3(f)). These findings indicated that PAR1 plays an important role in the downregulation of PTEN expression mediated by thrombin.

### 3.4. PTEN Plays a Role in Thrombin-Mediated Glc-82 Cellular Functions

PTEN, as a negative regulator of the PI3K/AKT pathway, plays critical roles in regulating cell growth, cell cycle, cell apoptosis, and cell migration [29]. Thrombin is also a key player in promoting cancer tumor growth, metastasis, and tumor-associated angiogenesis [3, 26, 30, 31]. To elucidate the effect of PTEN in thrombin-mediated Glc-82 cells functions, three tests (cell cycle, cell apoptosis, and cell migration) were performed in this study.

**Figure 4:**
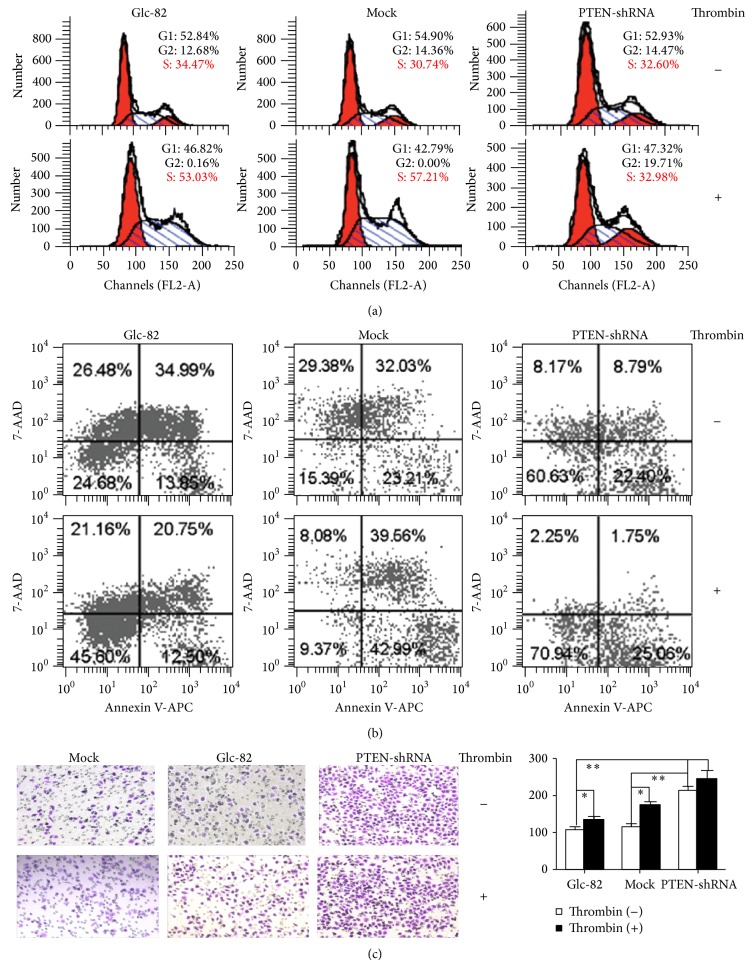
The role of PTEN in thrombin-mediated effects on Glc-82 cells. (a) The role of PTEN in thrombin-mediated promotion of Glc-82 cell cycle progression. Glc-82, Mock, and Glc-PTEN-shRNA cells were starved for 24 h in serum-free medium before treatment without or with thrombin (0.5 U/mL) for 12 h. The cell cycle was evaluated by flow cytometer analysis. (b) The role of PTEN in thrombin-mediated protection of Glc-82 cells from serum deprivation-induced apoptosis. Cells were starved for 48 h in serum-free RPMI 1640 medium. Thrombin (0.5 U/mL) was added and incubated for a further 96 h. The cells were then collected and analyzed on the flow cytometer within 1 h after staining. (c) The role of PTEN in thrombin-mediated promotion of Glc-82 cells migration. Cells were allowed to migrate for 20 h, cells in serum-free medium without or with thrombin (0.5 U/mL); then three different visual fields were randomly selected to count the cell number and calculate the average migration rate (^*^ *P* < 0.05, ^**^ *P* < 0.01).

As shown in Figure 4(a), thrombin significantly increased the number of cells in S phase by 18.56% in Glc-82 and by 26.14% in the Mock group. Compared with Glc-82 and Mock group, the number of cells in S phase in Glc-PTEN-shRNA cells was markedly deceased by 20.05–24.23% after thrombin treatment. This indicated that the effect of thrombin on Glc-82 cell cycle may be, at least partly, dependent on PTEN.

As shown in Figure 4(b), thrombin protected Glc-82, Mock, and Glc-PTEN-shRNA cells from late apoptosis induced by serum deprivation. Furthermore, the percentages of healthy cells and cells in an early apoptotic state were increased when PTEN was effectively silenced. This indicated that thrombin is a potent protective factor and that PTEN might be involved in the serum deprivation-induced apoptosis of Glc-82 cells.

Figure 4(c) showed that 0.5 U/mL thrombin significantly enhanced the migratory ability of Glc-82 and Mock cells, which agreed with a study showing that specific inhibition of thrombin leads to decreased tumor growth and metastasis [30]. The results suggested that PTEN also enhanced the migration capability, while thrombin treatment did not change this enhancement in Glc-PTEN-shRNA cells. These data suggested that thrombin promotes the migration of Glc-82 cells, perhaps involving PTEN.

## 4. Conclusions

Our findings indicated that thrombin promotes Glc-82 cells cycle progression and cell migration and inhibits cell apoptosis by downregulating PTEN expression, in association with activation of the PI3K/AKT signaling pathway. In addition, the thrombin receptor PAR1 is involved in the thrombin-mediated downregulation of PTEN. Therefore, a conceivable framework of the molecular mechanism of thrombin-induced lung cancer cell Glc-82 function could be as follows: thrombin activates PAR1 on the cell membrane; the activated PAR1 transfers some signal to downregulate PTEN expression; the negative regulation of PTEN on PI3K is attenuated, which activates the AKT signaling pathway and changes the expression of Skp2 and p27; finally, the cell function is affected by AKT signaling pathway. Thus, the present study revealed, for the first time, that the PTEN/PI3K/AKT signaling pathway plays an important role in thrombin/PAR1-mediated lung cancer cell growth and migration.

## Abbreviations Used

Mock: Short hairpin RNA expression plasmid as a control
PAR1: Protease-activated receptor 1
PI: Propidium iodide
PTEN: Phosphatase and tensin homolog deleted on chromosome 10
PTEN-shRNA: Short hairpin RNA expression plasmid targeting PTEN
shRNA: Short hairpin RNA
Skp2: S phase kinase associated protein 2.
